# The Role of Virgin Coconut Oil in Corn Starch/NCC-Based Nanocomposite Film Matrix: Physical, Mechanical, and Water Vapor Transmission Characteristics

**DOI:** 10.3390/polym15153239

**Published:** 2023-07-29

**Authors:** Heni Radiani Arifin, Fitriana Utaminingsih, Mohamad Djali, Bambang Nurhadi, Elazmanawati Lembong, Herlina Marta

**Affiliations:** Departement of Food Industrial Technology, Faculty of Agro-Industrial Technology, Universitas Padjadjaran, Sumedang 45363, Indonesia; fitrianau93@gmail.com (F.U.); djali@unpad.ac.id (M.D.); bambang.nurhadi@unpad.ac.id (B.N.); elazmanawati.lembong@unpad.ac.id (E.L.); herlina.marta@unpad.ac.id (H.M.)

**Keywords:** nanocrystalline cellulose, corn starch, nanocomposite film, virgin coconut oil

## Abstract

Corn starch-based nanocomposite films usually have low moisture barrier properties. Adding virgin coconut oil (VCO) as a hydrophobic component can improve the nanocomposite film’s characteristics, especially the film’s permeability and elongation properties. This study aimed to determine the role of VCO with various concentrations (0, 3, 5 wt%) on the physical, mechanical, and water vapor transmission characteristics of corn starch/NCC-based nanocomposite films. Adding 3% VCO to the film showed the lowest WVTR value by 4.721 g/m^2^.h. At the same time, the value of tensile strength was 4.243 MPa, elongation 69.28%, modulus of elasticity 0.062 MPa, thickness 0.219 mm, lightness 98.77, and water solubility 40.51%. However, adding 5 wt% VCO to the film increased the film’s elongation properties by 83.87%. The SEM test showed that adding VCO formed a finer structure with pores in several areas. The FTIR films showed that adding VCO caused a slightly higher absorption peak shift at the O–H groups and new absorption peaks at wave numbers 1741 cm^−1^ and 1742 cm^−1^. The results of this study may provide opportunities for the development of nanocomposite films as biodegradable packaging in the future.

## 1. Introduction

Plastic materials are the most widely used materials for packaging [1]. The drawback of plastic material for packaging is that it is difficult to decompose and is not renewable. The concerns for the environment and health encourage the development of biodegradable films derived from biopolymers, which decompose naturally, are renewable, and are environmentally friendly [2]. The potential of this biopolymer can be an alternative to reduce the consumption of synthetic plastics for food packaging [3]. Biopolymers are obtained from renewable resources such as plants, animals, microbes, and chemically synthesized polymers from naturally derived monomers [4].

Corn starch is a type of starch that contains a hydrocolloid component that can be used to form a film matrix [5]. The high amylose content of corn starch, around 25%, can create stronger films than starch which contains less amylose [6]. There is a shortage of starch as a film, which is brittle and has high water permeability, so there are still many limitations in the development of starch-based films as future packaging materials [7]. Efforts to improve the mechanical and barrier properties of the film require several combined approaches. The first approach is by adding nanocrystalline cellulose (NCC), commonly used to improve the mechanical properties of biopolymers [4]. These nano-sized particles have a high interaction surface area. The more particles that interact, the stronger the material [8]. However, to avoid aggregation in NCC during film synthesis due to high van der Waals attractions between nanoparticles, CMC (carboxymethyl cellulose) is needed as a compatibilizer which will reduce surface tension and increase interfacial adhesion between the polymer phase and the dispersed phase (NCC) to produce a dispersion that is homogeneous on the film matrix [9,10]. The second approach that can be conducted is to add hydrophobic molecules such as oils, fatty acids, and waxes to biopolymer-based films to improve the barrier properties of water vapor on the film [11]. Vegetable oils such as virgin coconut oil (VCO) can be added to the film solution because, at room temperature, its liquid nature makes it easier to combine with biopolymers [7].

VCO is rich in saturated fatty acids such as lauric acid and comprises approximately 90–95% of its saturated heavy acid content [12]. Saturated fatty acids exhibit lower molecular mobility than unsaturated fatty acids, reducing water vapor permeability [10,12]. In VCO, lauric acid can decrease water vapor permeability by forming amylose–lipid inclusion complexes, creating a compact network structure [13,14]. The VCO in the film acts as a water barrier and provides a tortuous effect on the diffusion path of the water vapor molecules, thereby reducing the film’s water vapor permeability [15]. However, as the concentration increases, network formation is found that is not formed/bound. If coconut oil is added at a large percentage, the coconut oil may experience leaching from the film [16]. According to Xiao et al. [17], the addition of VCO to the film has certain limits. A high concentration VCO addition induces phase separation during drying and failure to form a homogeneous film. The properties of water vapor transmission through emulsion or composite-based films depend on the crystal arrangement and length of the lipid chains, the dispersion of lipids in the matrix, and the type of surfactant used [18].

Several studies have reported the effect of lipid incorporation on nanocomposite films such as corn oil/gelatin [19] and bergamot oil/WPI [20] and discovered that it significantly improved the water vapor barrier properties of the films. However, nanoparticles have enhanced the water barrier property of the films less than oil. Oil-containing films had higher elongation at break and controversially lower tensile strength and modulus elasticity. However, the addition of lipids such as cinnamon essential oil/sugar palm starch [3] and olive oil/chitosan [21] reported that an increase in tensile strength and the addition of oil led to the elongation at the break of the films to decrease. It is strongly related to nanoparticle concentration rather than oil concentration. Several studies have reported the effect of VCO incorporations in the composite film matrix, such as potato starch [22], chitosan [23], and konjac glucomannan [17]. They found that VCO has been used to improve the water barrier properties of the film. The elongation at break values and flexibility of the film were considerably improved, even though the tensile strength of the composite films was marginally reduced at higher levels of VCO incorporation.

To our knowledge, research on adding VCO to nanocomposite films based on corn starch/NCC has never been carried out. The interaction of nanoparticles and lipids in the film matrix is possible and still needs to be explored. The combination of lipid components and NCC particles provides a more significant reduction in water vapor permeability compared to the addition of NCC alone [21]. Therefore, research on nanocomposite films reinforced with NCC with different VCO concentrations was conducted to determine the effect of optimal concentrations on nanocomposite films’ physical, mechanical, and water vapor transmission characteristics.

## 2. Materials and Methods

### 2.1. Materials

Corn starch (Maizenaku brand) was obtained from PT. Jaya Utama Santikah (Jakarta, Indonesia). Corn starch has an amylose content of 35.80%, amylopectin content of 46.34%, and Mw = 692.65802 g/mol. Virgin coconut oil/VCO (Vicoma brand) was from PT. Rachma Sari (Sukoharjo, Indonesia). The NCC is 413 nm (derived from Microcrystalline Cellulose Vivapur MCG 811 F was from JRS Pharma, Germany, processed by ball milling designed by PRINT-G laboratory at Universitas Padjadjaran, Bandung, Indonesia. Sorbitol (182.17 g/mol), carboxymethyl cellulose (CMC) (Mw = 265.204 g/mol, tween 80, filter paper, PP plastic, and silica gel (Wonder Natural brand) were obtained from Bratachem, Bandung, Indonesia.

### 2.2. VCO Emulsion Preparation

Preparation of VCO emulsions using an ultrasonicator refers to Gahruie et al. [24] with slight modifications. VCO with various concentrations (0, 3, 5 wt%) was put into the beaker glass. Furthermore, Tween 80, as much as 50% by weight of VCO, was added to the beaker glass. Then, both materials were mixed in 100 mL of distilled water and stirred using a stirrer for 5 min. The emulsion was then sonicated for 5 min with an amplitude of 65% to make it homogeneous. The emulsion formed was poured into the film solution.

### 2.3. Film Preparation

Nanocomposite films were made according to Arifin et al. [25] with modifications. An amount of 7 g of starch and 0.5% *w*/*v* CMC were dispersed in 85 mL of distilled water using a magnetic stirrer. Another beaker glass was prepared to disperse NCC as much as 5 wt% in 15 mL of distilled water and stirred until homogeneous. The dispersed NCC and the VCO emulsion were poured slowly into the starch solution while homogenizing. This dispersion was heated to a temperature of 62 °C, then 2% *v*/*v* sorbitol was added and continued to heat up to 80 °C, thus producing a film-forming material. Then, the film-forming material was treated with 3% or 5% VCO and sonicated at 40% amplitude for 10 min, while the 0% VCO treatment was not sonicated. The film-forming material was degassed in an ultrasound bath for 15 min. The nanocomposite dispersion film was poured into a glass plate mold with a size of 20 cm × 20 cm and dried at 45 °C for 20 h. The dried nanocomposite films were then removed from the mold.

### 2.4. Nanocomposite Film Characterization

#### 2.4.1. Film Thickness

Determination of the thickness of the nanocomposite film refers to the research method of Warkoyo et al. [26] by measuring the film using a micrometer with an accuracy of 0.01 mm. The test was carried out at five different points (four corners and center) then the results were calculated for the average thickness.

#### 2.4.2. Film Color

Determination of film color refers to Arifin et al. [25] who used a Spectrophotometer (CM-5, Konica Minolta Co., Osaka, Japan) with Software SpectraMagic NX Pro. Ver 2.6. Each sample was tested four times for an average color value (L*, a*, and b*). Calibration was performed with a blank calibration plate (CM-A124) and a white calibration plate (CM-A120). Measurements are taken by placing the film on the specimen holder and then shooting rays at two different points on the film.

#### 2.4.3. Film Solubility in Water

The determination of film solubility in water refers to Sahraee et al. [27]. The film samples were cut into squares with a size of 2 cm × 2 cm. Samples were dried at 105 °C in an oven for 24 h and then weighed as the dry weight of the film (mi). The dried samples were then immersed in 100 mL of distilled water at 25 °C for 24 h. The sample was filtered through filter paper and dried at 105 °C for 24 h to determine the remaining dry matter (md). Film solubility was calculated using the following formula.

(1) $$\%S=\frac{\left(mi-md\right)}{mi}\times 100\%$$

where S = film solubility (%); mi = the initial weight of the dry film (g); md = the weight of dry film residue after immersion (g).

#### 2.4.4. Water Vapor Transmission Rate of the Film

The nanocomposite films’ water vapor transmission rate was measured using the cup method based on ASTM E96-80 [28]. The test cup was filled with 10 g of silica gel. The sample was then cut according to the diameter of the surface of the cup to cover the cup, which already contained silica gel (RH = 0%). The cup was conditioned in a jar containing saturated NaCl solution (RH = 75%) at 25 °C. The cup was weighed every day, and the weight was measured. The data obtained was made into a linear regression equation, and the slope was determined. The slope was calculated from the absorption of the water vapor every hour. The following equation determined the water vapor transmission rate (WVTR):

(2) $$WVTR=\frac{slope}{A}$$

where WVTR = the water vapor transmission rate (g/m^2^.h); slope = absorption of water vapor per hour (g/h); A = the area of the film (m^2^).

#### 2.4.5. Mechanical Properties of the Film

The determination of mechanical characteristics refers to ASTM D882-10 [29] using the Universal Testing Machine (UTM) (Shimadzu AGS-X Series, Shimadzu Europa GmbH, Duisburg, Germany) with a load cell of 10 kN and a speed of 10 mm/min. The film samples were cut rectangularly with a size length of 8 cm and width of 2 cm. Samples for mechanical characteristic testing were previously conditioned at a controlled temperature of ±23 °C at around 50% RH for at least 24 h to reach a moisture balance point. These mechanical characteristics include tensile strength, elongation, and modulus of elasticity. Mechanical characteristics are determined using the following formula:

(3) $$\sigma =\frac{F}{A}$$

(4) $$\varepsilon =\frac{l-lo}{lo}\times 100$$

(5) $$E=\frac{\sigma }{\varepsilon }$$

where σ = the tensile strength (N/mm^2^); A = the area of the working force (mm^2^); F = the tensile force (N); ε = elongation (%); l = the final length at the time of damage to the sample (cm); lo = the initial length of the sample (cm); E = the modulus of elasticity (MPa).

#### 2.4.6. Fourier Transform Infrared Spectroscopy (FTIR)

FTIR functional group analysis refers to Setiani et al. [30] using the FTIR spectrophotometer (Thermo Scientific R Nicolet iS5, Thermo Fisher Scientific Inc., Waltham, MA, USA) with detector DGTS (Deuterated Triglycine Sulfate), ZnSe iD3 ATR Holder). The film sample was placed into the set holder, and then the appropriate spectrum was searched in wave number 500–4000 cm^−1^. Functional groups were identified by analyzing the infrared spectrum according to the peaks and wave numbers formed using the table of functional groups.

#### 2.4.7. Scanning Electron Microscope (SEM)

The determination of film morphology refers to Febriati [31] using a Scanning Electron Microscope (JEOL JSM-6360LA, JEOL Ltd., Tokyo, Japan). The film sample was cut to 2 cm × 2 cm, affixed to the set holder, and then coated with gold metal. The sample was then observed on topographical images with several magnifications.

#### 2.4.8. Statistical Analysis

The data were analyzed by One-way Analysis of Variance (ANOVA) using the software SPSS Statistic Ver 25.0 to compare VCO concentrations in the nanocomposite film and submitted to the mean comparison test by Duncan at a significance level of 5% (*p* < 0.05) to determine the significant difference between treatments.

## 3. Results and Discussion

### 3.1. Film Thickness

The thickness of the nanocomposite film produced in this study can be seen in Table 1. The thickness of the nanocomposite film increases with an increasing VCO concentration. The 5 wt% VCO treatment resulted in the thickest nanocomposite films. The 3 wt% and 5 wt% VCO treatments experienced an increase in thickness of 2% and 10.6% from the 0 wt% treatment, respectively. However, the values of all the treatments were not significantly different. The thickness of the nanocomposite film could be affected by the material’s concentration, the mold’s area, and the volume of the solution poured into the mold [24,25]. The molds and the film solution volume used to form nanocomposite films were the same. In total, 160 mL of film solution was poured into a 20 cm × 20 cm mold for all treatments. Different concentrations of materials had more effect on thickness differences in this study. This was caused by the presence of chemical components found in VCO, which could interact with starch to form agglomerates, thus giving a prominent structure to the film [3].

Thickness is an important characteristic in biodegradable films because it affects the characteristics of the packaging material, such as tensile strength, percent elongation (elongation), and water vapor and gas permeability [32]. Based on the plastic film standard in the JIS Standard [33], the maximum film thickness value was 0.25 mm. The thicker the nanocomposite film, the stiffer and harder the film will be, which will increase the safety of the packaged product because, usually, the film has a strong film matrix. The nanocomposite films from all treatments met the food packaging film thickness standards.

### 3.2. Film Color

**Figure 1:**
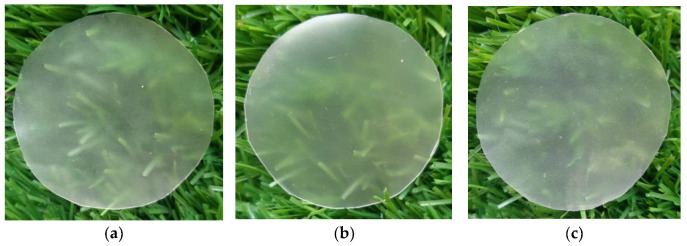
The appearance of nanocomposite films with (**a**) 0 wt%; (**b**) 3 wt%; and (**c**) 5 wt% of VCO.

The color parameters in the form of L*, a*, and b* nanocomposite film values are shown in Table 1. Meanwhile, the appearance of the film can be seen in Figure 1. Based on Table 1, the average L* nanocomposite film value had an increasing lightness level. A high L* value decreased the nanocomposite film’s a* and b* values. None of the L* values were significantly different. The addition of components, in general, caused discoloration of the film. Moreover, if the components included in the film solution have a color [34]. The results of this study were not in line with Ghasemlou et al. [35] which stated that the film added to the oil emulsion would increase the reflectance diffusion by light scattering in the lipid droplets, reducing the intensity of the light transmitted to the film or more opaque, and the L* value decreased. This study gave the opposite results, namely an increase in the value of L* as the concentration of VCO increased. This may occur because oil causes the polymer matrix to become less compact and could also prevent starch from crystallizing. This crystallization could reduce the transparency and gloss of the film. Therefore, the presence of oil could reduce crystallization and make the film more translucent [36].

The film color parameters are shown in Table 1, which shows that the value of a* in this study increased with increasing VCO concentration. A positive value of a* indicated a tendency for a red color. The a* value of the VCO film was significantly higher than the film without VCO addition. Meanwhile, the value of b* showed fluctuations with increasing VCO concentration. A positive value of b* indicated that the film tends to be yellow [37]. The results of this study were slightly different from the study by Binsi et al. [23] which stated that adding VCO to chitosan-based films gave a negative value of a*, which indicated a tendency for the film to be green. Differences in the color tendency of the films may occur due to differences in the composition of the film constituents. The increase in b* value on adding 5 wt% VCO occurred because the concentration of VCO was higher, so the film tended to be yellow. The type of surfactant could also affect the color of the film. Films with the addition of yellow tween 80 gave a relatively high b* value [38]. The 5 wt% VCO treatment in this study contained more tween 80 than the other treatments. This causes the b* value to be even higher. Furthermore, the 5%wt VCO film was significantly different from other treatments. From a visual point of view, this film did not have a big difference.

### 3.3. Film Solubility in Water

The solubility of the nanocomposite films produced in this study is shown in Table 2. Nanocomposite films treated with 0 wt% VCO had the highest solubility in water. Adding 3 wt% and 5 wt% VCO decreased the solubility of the films by around 0.5% and 1.7% compared to the 0 wt% treatment, respectively. However, not all of the treatment significantly decreased. The decrease in the solubility of the film in water was due to the presence of fatty acids, which had hydrophobic properties in the VCO, so the film was difficult to dissolve in water [17]. The ability of the film to dissolve in water was affected by the length of the fatty acid carbon chains. The longer the carbon chain, the more difficult it was for the fatty acids to dissolve in water [39]. VCO had the most lauric acid composition. Lauric acid is a medium-chain fatty acid with 12 carbon atoms [40].

The results showed that incorporating VCO in the nanocomposite film affected the film solubility. These results were in line with Sahraee et al. [19], which stated that increasing the concentration of lipids on the polymer matrix increased the hydrophobic density and could reduce the tendency of the film to absorb water molecules. Therefore, the 5 wt% treatment had the lowest solubility compared to other treatments. The decrease in film solubility in water could also occur due to a synergistic effect between the hydrophobic components (VCO) and nanoparticles (NCC). Nanoparticles could form more hydrogen bonds with the polymer matrix so water molecules could not bind to these nanoparticles. This could increase the cohesion between film components which causes reduced hydroxyl groups to bind with water [25]. Meanwhile, on the other hand, the hydrophobic component reduced the availability of polymer hydroxyl groups to bind with water. The results of the interaction of the two components reduced the solubility of the film in water [41].

### 3.4. Water Vapor Transmission Rate of the Film

The water vapor transmission rate (WVTR) of the nanocomposite films produced in this study is presented in Table 2. The transmission rates of the nanocomposite films decreased in the 3 wt% treatment by around 5.2% from the 0 wt% treatment. The increase occurred when 5 wt% VCO was added, approximately 6.8% of the 0 wt% treatment. However, the film was not significantly different at each concentration of VCO. Based on the JIS Standard [35], the maximum value for WVTR was 10 g/m^2^·h. The WVTR value of the nanocomposite films in all treatments in this study met the standards for food packaging because they were able to inhibit the amount of water vapor entering or leaving through the film, thereby protecting food from the growth of microorganisms.

The treatment of nanocomposite films with 3 wt% VCO had the lowest water vapor transmission rate compared to other nanocomposite films. This was presumably caused by the fatty acids presented in VCO increasing the hydrophobic properties of the film, thereby increasing the water barrier properties [15]. In addition, the high amylose content in corn starch positively correlated with the amount of starch–lipid inclusions. This amylose was capable of forming helical inclusion complexes [42]. Starch granules heated to the gelatinization temperature in water would expand due to loss of the crystal structure and water absorption. At this time, the amylose in the starch granules shaped amylose–lipid complexes with fatty acids. The ends of the lipid carbon chains were located in the helix of the amylose molecule, and this combination shaped an inclusion complex [43]. Sincerely increasing the VCO content made starch–lipid inclusions form more efficiently. This caused the formation of a denser network structure, thereby strengthening the hydrophobic properties of starch-based films. So, water molecules would find it difficult to penetrate the starch layer and reduce the WVTR value [22]. In addition, the water vapor transmission rate could also be related to the film thickness. The thicker the film produced, the higher its ability to inhibit the rate of gas and water vapor [44].

Besides that, the treatment of nanocomposite films with 5 wt% VCO had the highest water vapor transmission rate. This was presumably due to a high concentration of hydrophobic components, which could disrupt the internal network of the film by forming a porous structure and opening up the polymer matrix because the bonds between covalent and non-covalent bonds were weak, allowing water molecules to pass easily through the film matrix [20]. Besides that, the migration of lipid droplets at higher concentrations during the drying process caused the film matrix to become uneven and formed a porous surface. In addition, most of the solutions were made of hydrophilic starch. The hydrophobic to hydrophilic ratio of the film increased with the addition of oil. Still, the hydrophilic properties of other components may exceed the ratio, increasing the water vapor passing through the film [45].

Furthermore, the role of lipids in WVTR depended on the type of lipids, their compatibility, preparation techniques, and their distribution in the polymer matrix [46]. In this study, lipids in the form of VCO had a plasticizing effect, allowing them to open the matrix, thereby increasing the diffusion of water molecules. On the other hand, forming a lipid network in the film matrix created a tortuous path to decrease the diffusion of water molecules. Therefore, the final effect of lipids on WVTR depended on the dominant mechanism [15].

### 3.5. Mechanical Properties of the Film

The mechanical characteristics of the films produced in this study are shown in Table 3. The addition of VCO concentration decreased the tensile strength of the nanocomposite films. The nanocomposite films experienced a decrease in tensile strength of about 11.6% when 3 wt% VCO was added. Similar values were obtained when 5 wt% VCO was added, with a reduction of approximately 0.14% between the two treatments. The highest VCO concentration resulted in the lowest tensile strength value, although the results were nearly identical with the concentration of 3 wt%. Increasing the VCO content did not significantly reduce the nanocomposite films’ tensile strength. Based on the JIS Standard [33], the minimum value for tensile strength was 0.3 MPa, so the nanocomposite films in all treatments in this study met the standards and were suitable for food packaging because of their strong structure.

The decrease in tensile strength after adding VCO (3 and 5 wt%) was presumably due to the reduced interaction between the polymer matrices. Increasing the VCO concentration increased the contact between lipid and starch molecules, which resulted in more significant lipid discontinuity in the polymer matrix [23]. Reducing hydrogen bonds and decreasing intermolecular forces reduced the polymer’s cohesiveness and the film’s tensile strength [22]. The decrease in tensile strength in this study was in line with Zhang et al. [47], who stated that the combination of lipids and polymers replaced the strong polar bonds in the chitosan polymer, thus making the film matrix structure heterogeneous and discontinuous, resulting in decreased tensile strength. In addition, adding VCO will also increase the thickness of the film, so this increase reduces the film’s tensile strength [23]. Tensile strength provides an interaction that is inversely proportional to the thickness of the film.

Moreover, this research combines the matrix and VCO dispersion phases in a mixed structure. The volume percentage of the dispersed phase determines the tensile strength characteristics of the non-adhesive hybrid system [16]. Besides VCO, the presence of tween 80 also affects the film’s tensile strength. Tween 80 was a hydrophilic surfactant with a high hydrophilic–lipophilic (HLB) balance that could interact with water or starch. These interactions could damage intermolecular hydrogen bonds, reducing mechanical properties such as tensile strength [38].

Based on Table 3, the film elongation value appears to have a fluctuating trend. There was a decrease in elongation of about 14.1% from the control treatment to the 3 wt% VCO treatment. At the same time, the increase in elongation from the 0% wt to the 5 wt% VCO treatment occurred around 5.04%. The 3 wt% VCO treatment experienced a decrease in elongation, which might be caused by less homogeneous mixing so that the VCO and plasticizer act as a plasticizer, the insertion process into the film matrix could have been better, and the resulting elongation was not optimal. Furthermore, the elongation of the 3 wt% treatment showed a significant reduction compared to other treatments. The 5 wt% VCO treatment experienced an increase in elongation because oil, in this case, VCO, could act as a plasticizer in the hydrocolloid network, which could increase film flexibility [23].

VCO was liquid at room temperature and was in the form of oil droplets in the composite film. These oil droplets were easily deformed, which could increase the plasticity of the film [38]. More oil in the 5 wt% VCO treatment also resulted in weak polymer–oil interactions, replacing some of the stronger polymer–polymer interactions in the film network. Film elongation could also increase with this phenomenon [44].

Meanwhile, plasticizers could reduce the cohesion between the mechanical bonds in the polymer and change the film’s density to become more flexible. High tensile strength would have a low elongation value at the breaking point because the film was difficult to break and had lower flexibility [23]. Based on the JIS Standard [33], elongation values above 50% were considered good characteristics. All nanocomposite film treatments in this study had elongation values above 50%, with the lowest value being 61.18%. All film treatments showed quite high elastic properties.

Based on Table 3, the value of the film elasticity modulus appeared to have a fluctuating trend. The highest concentration of VCO reduced elasticity up to 0.051 MPa. No significant difference was detected in the modulus elasticity of the films with and without the incorporation of VCO. Higher VCO concentrations could increase film elongation because lipids could act as plasticizers that weaken polymer bonds [48]. Weak polymer bonds would become more mobile so that the elongation of the film would increase, and the elastic modulus would decrease compared to the 3 wt% VCO treatment [17]. Adding higher VCO caused the film to be more elastic, and the modulus of elasticity decreased. Meanwhile, in the 3 wt% VCO treatment, there was an increase in the modulus of elasticity which might have occurred because inserting the plasticizer into the film matrix had not been perfect during the stirring process. The results of this study differed from Syafiq et al. [3], which stated that adding a hydrophobic material in the form of cinnamon essential oil to the film matrix increased the film’s stiffness. Hence, the modulus of elasticity also increased.

### 3.6. Scanning Electron Microscopy (SEM)

**Figure 2:**
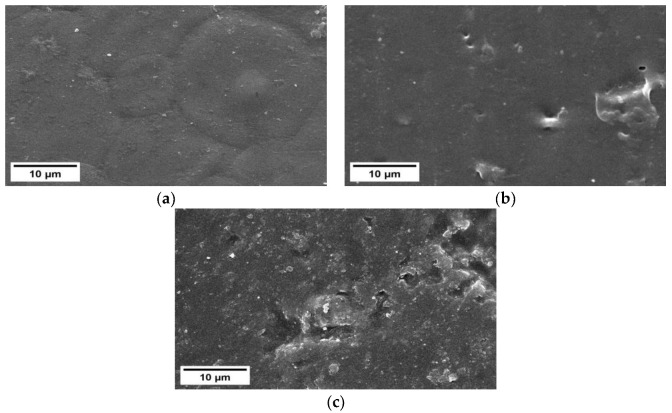
Nanocomposite film morphology with the addition of VCO at a concentration of (**a**) 0 wt%; (**b**) 3 wt%; and (**c**) 5 wt% with a magnification of 2500×.

The appearance of the surface of the nanocomposite film is presented in Figure 2. The 0 wt% VCO treated film showed an irregularly rounded surface and looked rough without forming pores. The nanocomposite film with the addition of 3 wt% VCO looked smooth, but there were pores of different sizes in some areas. Adding more VCO to the 5 wt% VCO treatment increased the rough surface of the film and formed cavities. This result might be due to the weak interaction between the film matrix’s oil and polysaccharides [40]. During drying, lipid destabilization occurred and caused lipid droplets to move toward the film’s surface [3]. These lipid droplets formed cavities throughout the matrix. This disability phenomenon made the film matrix structure inhomogeneous and formed a porous surface [17]. This unstable texture with lots of pores and holes could have a negative impact on the mechanical and barrier properties of the film produced [15]. The more porous structure in the 5 wt% VCO treatment caused a higher molecular diffusion. This was also seen in the WVTR of the 5 wt% VCO treatment, whose value increased.

Using an emulsifier as tween 80 was expected to form a homogeneous and stable solution to prevent recombination during film preparation or drying [49]. However, this seemed not to have happened because of the pores or cavities in the resulting film. The possibility of pore formation was due to the ultrasonic energy, which was too large, so the treatment damaged the polymer structure to a certain extent [50].

### 3.7. Fourier Transform Infrared Spectroscopy (FTIR)

**Figure 3:**
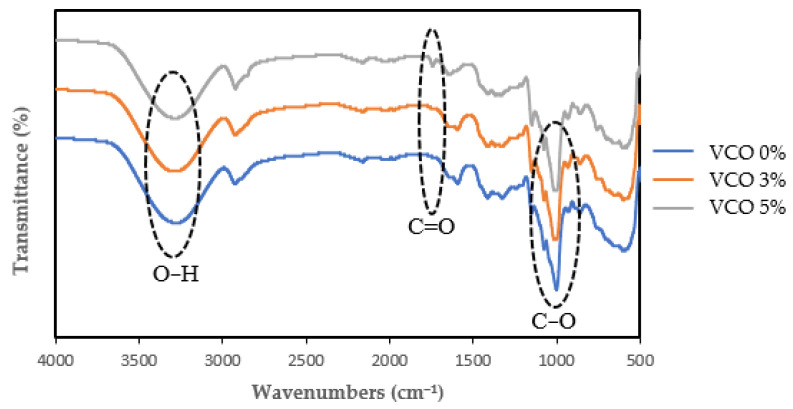
FTIR absorption of nanocomposites films.

The FTIR absorption of nanocomposite films is shown in Figure 3. The broad absorption peak at 3000–3700 cm^−1^ indicated the presence of O–H groups. It could be seen that there was a broad absorption peak located at 3277–3285 cm^−1,^ which was the O–H stretching group. Functional group analysis with FTIR aims to determine the processes of mixing the ingredients and compare the functional groups in the films produced with each treatment. The addition of 3 and 5 wt% VCO caused a slightly higher shift in absorption peak at 3280 cm^−1^ and 3285 cm^−1^, compared to the 0 wt% VCO film, 3277 cm^−1^. This indicated the presence of VCO-containing hydrocarbons in the matrix. The increase in wavelength could occur due to differences in the conformation of the molecular structure due to the addition of VCO [3].

In addition, treatment with 3 wt% and 5 wt% VCO showed absorption peaks at wave numbers 1741 cm^−1^ and 1742 cm^−1^, respectively, while in the 0 wt% VCO treatment, there was no absorption at that wave number. Wavenumbers greater than 996 cm^−1^ were associated with C–O stretching in the glycosidic bond. The presence suspected of a stretching carbonyl group (C=O) of the fatty acid ester component was appropriate because the 0% VCO treatment did not add lipids [17].

There were absorption peaks at wave numbers 1000, 1013, and 1012 cm^−1^ for the 0 wt%, 3 wt%, and 5 wt% VCO treatment, respectively. These results allegedly indicated a C–O group from the polysaccharide and sorbitol components. As substances were mixed, the physical mixture reacted chemically to cause changes in spectral peaks [3]. The presence of new absorption peaks, the shift in absorption peaks, and differences in transmittance acuity are thought to be due to interactions between VCO, starch, and NCC. The spectrum of the nanocomposite film is summarized in Table 4.

## 4. Conclusions

This study concluded that differences in VCO concentrations result in interactions between all components, such as plasticizers, nanoparticles, surfactants, and polymer matrices which affected the characteristics of the nanocomposite films. Lipids’ role in this barrier film’s characteristics depended on the type of lipids, their compatibility, and their distribution in the polymer matrix. There were two possibilities of VCO affecting the barrier film characteristics. In this study, lipids in the form of VCO had a plasticizing effect, allowing it to increase the diffusion of water molecules. On the other hand, the formation of lipid networks in the film matrix could also create tortuous pathways to decrease the diffusion of water molecules. Although the WVTR value was not significantly different, adding 3 wt% VCO could reduce the value of WVTR. This treatment had the lowest WVTR. This treatment produced nanocomposite film characteristics with an average thickness of 0.219 mm, brightness of 98.77, solubility in water of 40.51%, tensile strength of 4.243 MPa, elongation of 69.28%, modulus of elasticity of 0.062 MPa, and WVTR of 4.721 g/m^2^·h. At the same time, adding 5 wt% VCO could increase the elongation properties of the film according to JIS standards. Therefore, this film has the potential to be developed into biodegradable packaging. In the future, it is necessary to research added variations in VCO concentration treatments to obtain an optimum decrease in WVTR and increase in elongation film.

## Figures and Tables

**Table 1:** Thickness and color parameters of nanocomposite films with the addition of different concentrations of VCO.

Treatment	Thickness (mm)	L*	a*	b*
VCO 0 wt%	0.214 ± 0.02 ^a^	98.55 ± 0.34 ^a^	0.11 ± 0.04 ^b^	1.63 ± 0.15 ^b^
VCO 3 wt%	0.219 ± 0.02 ^a^	98.77 ± 0.26 ^a^	0.23 ± 0.05 ^a^	1.60 ± 0.05 ^b^
VCO 5 wt%	0.237 ± 0.01 ^a^	98.98 ± 0.15 ^a^	0.30 ± 0.04 ^a^	1.90 ± 0.02 ^a^

L* = lightness; a* = chromatic green-red color; b* = chromatic blue-yellow color wt% (Based on corn starch). According to the Duncan test, the mean treatment value denoted by the same letter indicates no significant difference at the 5% test level.

**Table 2:** Film solubility in water and WVTR of nanocomposite films with the addition of different concentrations of VCO.

Treatment	Film Solubility in Water (%)	WVTR (g/m^2^·h)
VCO 0 wt%	40.71 ± 2.39 ^a^	4.982 ± 0.10 ^a^
VCO 3 wt%	40.51 ± 0.73 ^a^	4.721 ± 0.53 ^a^
VCO 5 wt%	40.02 ± 0.84 ^a^	5.324 ± 0.59 ^a^

According to the Duncan test, the mean treatment value denoted by the same letter indicates no significant difference at the 5% test level.

**Table 3:** Tensile strength, elongation, and modulus elasticity of the film.

Treatment	Tensile Strength MPa	Elongation (%)	Modulus Elasticity MPa
VCO 0 wt%	4.800 ± 0.73 ^a^	79.84 ± 2.07 ^a^	0.060 ± 0.01 ^a^
VCO 3 wt%	4.243 ± 0.3 ^a^	69.28 ± 1.83 ^b^	0.062 ± 0.01 ^a^
VCO 5 wt%	4.237 ± 0.36 ^a^	83.87 ± 5.85 ^a^	0.051 ± 0.01 ^a^

According to the Duncan test, the mean treatment value denoted by the same letter indicates no significant difference at the 5% test level.

**Table 4:** FTIR Spectrum Wavenumber of Nanocomposite Film.

Wave Number (cm^−1^)	Absorption Peak	References
3000–3700	O–H group	[17]
1000–1200	C–O	[51]
940–1200	C–C	[51]
~995 cm	C–O stretching vibrations	[3]

## Data Availability

Not applicable.
